# Influence of the Probiotic *L. reuteri* on Periodontal Clinical Parameters after Nonsurgical Treatment: A Systematic Review

**DOI:** 10.3390/microorganisms11061449

**Published:** 2023-05-30

**Authors:** Carlota Ochôa, Filipe Castro, José Frias Bulhosa, Conceição Manso, Juliana Campos Hasse Fernandes, Gustavo Vicentis Oliveira Fernandes

**Affiliations:** 1 FP-I3ID, FCS, Fernando Pessoa University, 4249-004 Porto, Portugal; 2 Periodontics and Oral Medicine Department, University of Michigan School of Dentistry, Ann Arbor, MI 48109, USA

**Keywords:** periodontal disease, probiotics, nonsurgical treatment, *L. reuteri*

## Abstract

The aim of this systematic review was to evaluate the actual efficacy of *Lactobacillus reuteri* (*L. reuteri*) on the periodontal clinical parameters when used concomitantly to the nonsurgical periodontal treatment. Searches were conducted through PubMed Central, Online Knowledge Library, Science Direct, Scielo, and Cochrane databases from 2012 to 2022. The focused question was “In patients with periodontitis, will the probiotic *L. reuteri*, when administrated as an adjunct to nonsurgical periodontal treatment, compared to the nonsurgical periodontal treatment alone, result in better clinical outcomes?” The following information was extracted from the articles: author and year of publication, type of study, follow-up, sample size and number of defects, and clinical characteristics and details. All included studies were qualitatively assessed using the Critical Appraisal tools according to the Joanna Briggs Institute. Twenty-four articles were full-text reading, but only 9 articles were included. The number of patients enrolled was 287, aged between 18 and 56 years. All periodontal parameters were evaluated. The “follow-up” varied (14, 40, 84, 90, 180, and 360 days). Most articles supported the clinical benefits of *L. reuteri* as an adjunct to SRP compared to SRP alone. A common finding at the beginning period was thatno statistically different results were observed between the test and control groups; otherwise, at the last period, a significant improvement was found in favor of the probiotic use (*p* = 0.001) for all the clinical parameters. The use of *L. reuteri* as an adjunct to nonsurgical periodontal treatment may result in significantly better clinical outcomes than nonsurgical periodontal treatment alone; but the conclusion must be carefully interpreted because of the heterogeneity found among the studies.

## 1. Introduction

The oral cavity is an environment with several niches (saliva, gingival fluid, and epithelial or mineralized surfaces) that house numerous bacteria. They interact in a homologous pathway, in the case of health or dysbiosis when associated with disease [1]. Periodontal disease is an inflammatory problem due to specific pathogens from plaque accumulation. Initially, it affects the gingiva (reversible lesion, gingivitis). In a more advanced stage, it can jeopardize the tissues of support (bone, periodontal ligament, and cementum) [2], and be characterized by periodontitis (non-reversible condition).

According to the European Federation of Periodontology (EFP), periodontitis was defined as a chronic multifactorial inflammatory disease associated with bacterial dysbiosis and characterized by the progressive loss of supporting tissues of the tooth. It is the 6th most frequent pathology in the world and is the 2nd leading cause of tooth loss worldwide [3]. Its prevalence achieves 47.2% in adults aged 30 or older, affecting more men (56.4%) than women (38.4%), according to the Centers for Disease Control and Prevention (CDC) [4].

Within this scenario, nonsurgical periodontal therapy has importance. It aims to reduce the pathogens through supra and subgingival instrumentation, performing scaling and root planing (SRP) in pockets ≥ 4 mm, allowing a mechanical removal of biofilm, in addition to enhance the motivation and oral hygiene instructions. Its primary goal is to decrease the probing pocket depth (PD), plaque index (PI), and bleeding on probing (BoP) [5,6]. Antibiotic therapy can be implemented in cases of advanced periodontitis and recurrent deep pockets after SRP. The most used are Amoxicillin and Metronidazole [7].

Generally, anaerobic gram-negative bacteria are more sensitive to Metronidazole. However, *A. actinomycetemcomitans* (A.a.) is more sensitive to Amoxicillin and more resistant to Metronidazole. Thus, implementing the association (Amoxicillin with Metronidazole) creates more favorable clinical and microbiological effects than Metronidazole alone, despite presenting more adverse effects [8]. The greatest question is cases of microbial resistance [9], recolonization in the same sites, and possible adverse effects [10,11].

In this regard, the use of probiotics [11] in periodontitis treatments has received attention as a possible modifier in the pathogenic-bacterial composition, even though it is temporarily used or combined with antisera or antibiotics [12]. The administration of probiotics as an adjuvant to periodontal treatment has improved periodontal clinical parameters, significantly decreasing the concentration of periodontal pathogens without causing any side effects [13]. The World Health Organization (WHO) and the Food and Agriculture Organization (FAO) characterize probiotics as live microorganisms which, when administered in optimal and adequate amounts, are beneficial to the individual’s overall health [14]. Recently, there has been a growing interest in using probiotics for oral health [15]. They stimulate the immune system of the oral mucosa by reducing the production of pro-inflammatory cytokines and increasing the production of anti-inflammatory cytokines.

The main pathways in which probiotics act: (i) producing antibacterial substances against periodontopathogens; (ii) performing innate and acquired modulation of host defense (increased activity of natural killer [NK] cells); and finally, (iii) increasing the number of beneficial bacteria to delay recolonization of the periodontopathogenic group [16]. The probiotics most used, by evidence from several studies, are *Lactobacillus reuteri*, *Bifidobacterium* spp., *Lactobacillus plantarum*, *Lactobacillus brevis*, and *Lactobacillus rhamnosus*. However, the most commonly used probiotic among those mentioned above, which is currently used as adjunctive in periodontal treatment after SRP, especially in deep pockets, is *Lactobacillus reuteri* [6].

*L. reuteri* has antibacterial [17] and immuno-inflammatory properties (reduction of metalloproteinase-8 [MMP-8] in gingival crevicular fluid) when used in the treatment of periodontitis. In addition, it has the ability to prevent pathogenic microorganisms’ growth [2]. This probiotic is a heterofermentative bacterium that has two strains: (1) DSM 17938, which acts as an antibiotic-producing reuterin, inducing oxidative stress on pathogens; and (2) ATCC PTA 5289 with anti-inflammatory characteristics, producing TNF, IL-8, and IL-1beta [18].

Thus, the objective of this systematic review was to understand the actual efficacy of *Lactobacillus reuteri* (*L. reuteri*) on the periodontal clinical parameters when used concomitantly to the nonsurgical periodontal treatment. The primary outcomes were pocket depth (PD), bleeding on probing (BOP), and clinical attachment level (CAL); the secondary outcomes observed were plaque index (PI), gingival index (GI), gingival bleeding index (GBI), and recession (REC). The positive hypothesis is that the adjunctive use of *L. reuteri* significantly improves the results in periodontal treatments.

## 2. Materials and Methods

This study was prepared according to the PRISMA (Preferred Reporting Items for Systematic reviews and Meta-Analyses) guidelines [19,20]. The focus question was designed based on the PICO (P = Population; I = Intervention; C = Comparison; O = Results) strategy (Table 1): “Will the probiotic *L. reuteri*, when administrated and used as an adjunct to nonsurgical periodontal treatment, result in better clinical outcomes compared to the nonsurgical periodontal treatment alone?”

### 2.1. Study Selection and Eligibility

Based on the objectives outlined, the search of scientific articles was done in PubMed Central (PMC), Online Knowledge Library (B-On), Science Direct, Scielo, and Cochrane Library, between June 2022 and October 2022. It used the following keywords: “*periodontal disease*”, “*nonsurgical treatment*”, “*probiotics*”, and “*L. reuteri*” associated with BOOLEAN markers “AND” and “OR”, with specific adjustments for each database. The research was subjected to inclusion and exclusion criteria (Table 2). Three independent reviewers (CO, FC, JFB) performed the appraisal. The reviewers discussed the results based on the inclusion/exclusion criteria, observing first the title and abstract. Subsequently, the studies that met the inclusion criteria or those with insufficient data in the abstract to make a clear decision were selected to evaluate the entire manuscript. Duplicate articles were removed. Only English-language articles, articles published in the last 10 years (December 2012 to December 2022), and those presenting information relevant to the topic under study were selected.

### 2.2. Selection of Articles and Data Extraction

The studies that met the inclusion criteria or those with insufficient data in the abstract to make a clear decision were selected to evaluate the full manuscript. The following information was extracted from the articles: (i) author and year of publication; (ii) type of study; (iii) follow-up; (iv) sample size and the number of defects; and (v) clinical characteristics and details (clinical attachment level [CAL] gain, pocket depth [PD] reduction, and recession [REC] reduction).

### 2.3. Risk of Bias

All included studies were qualitatively assessed. Two independent investigators (CO and FC) performed the quality assessment. In the case of divergences, a third researcher was consulted (GVOF). It used the Critical Appraisal tools, according to the Joanna Briggs Institute (JBI) for Systematic Reviews, to determine the extent to which a study has addressed the possibility of bias in its design, conduct, and/or analysis. Seven main topics were approached in 12 questions: (i) sequence generation; (ii) allocation concealment; (iii) blinding of participants and personnel; (iv) blinding of outcome assessors; (v) incomplete data; (vi) selective outcome; and (vii) other sources of bias (such as funding or conflict of interest).

The risk of bias in the included studies was categorized as below: (a) low risk of bias (plausible bias unlikely to seriously alter the results) if all criteria were met (all green [yes]) or at maximum 2 were unclear; (b) moderate risk of bias (“plausible bias” data raises some doubt about the results) if one “no” (red) is found or up to 4 “unclear” criteria were met; (c) high risk of bias (plausible bias that seriously weakens confidence in the results) if one or more criteria were not met (at least 2 “no” (red) or ≥5 “unclear” is found); and (d) risk evaluation not applicable to this context.

## 3. Results

**Figure 1:**
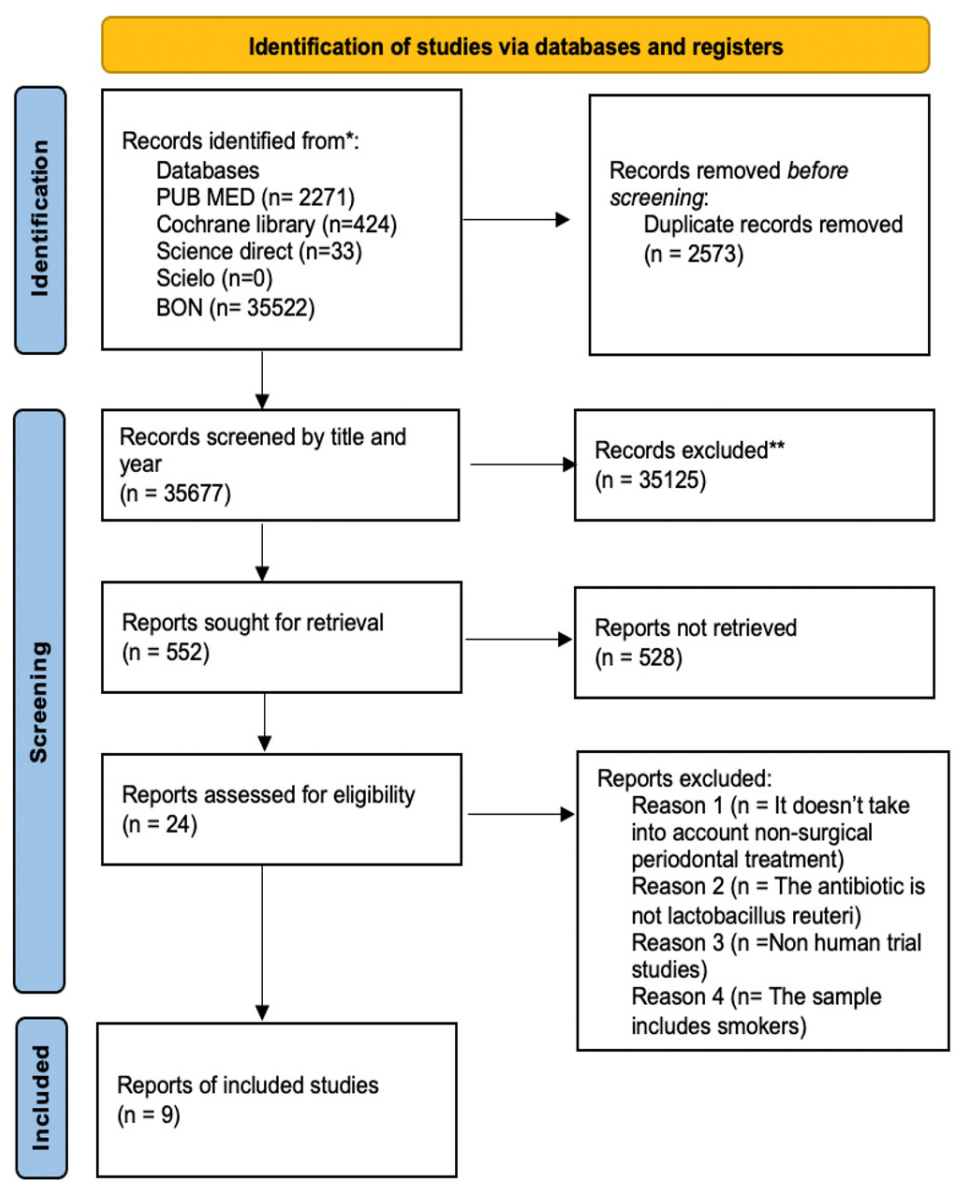
Flowchart with the different research phases and study selection process. * Databases used to screen and record studies; ** Records excluded in the first-round reading.

In the identification phase, 38,250 articles were obtained, of which 35,677 were removed for duplication (*n* = 2573). In the selection phase, 35,125 were eliminated for having titles that were not relevant; consecutively, only 552 articles were relevant. After reading the abstract, 528 articles were eliminated, resulting in 24 articles being analyzed in full by the three authors (*k* = 0.93). Of the 24 articles analyzed, 4 articles were excluded because they did not address nonsurgical periodontal treatment, 8 because the *L. reuteri* strain was not used, 1 article was an in vitro study, and finally, 2 articles included patients who smoked. Then, the literature search on the influence of *L. reuteri* in the nonsurgical treatment of periodontal disease resulted in 9 articles considering current scientific evidence, which met the inclusion and exclusion criteria, and were integrated in this systematic review (*k* = 0.99) (Figure 1). They were codified to facilitate the description: S1 (Teughels et al., 2013) [21]; S2 (Tekce et al., 2015) [22]; S3 (Pelekos et al., 2019) [15]; S4 (Ikram et al., 2019) [23]; S5 (Sinulingga et al., 2020) [10]; S6 (Pelekos et al., 2020) [24]; S7 (Hadžić et al., 2021) [25]; S8 (El-Bagoory et al., 2021) [26]; and S9 (Sufaru et al., 2022) [27].

### 3.1. Demographic Data and Oral Hygiene Recommendations

A total of 287 patients (145 female and 126 males; 16 patients did not have their gender identified) were enrolled and were not allowed to use any antibiotic therapy during the study period; only systemically healthy individuals were included (Table 3). Age ranged from 18 to 56 years, and the number of patients varied between 12 and 40. Then, most of the articles supported the clinical benefits of administering *L. reuteri* as an adjunct to SRP when compared to SRP alone.

In most articles, oral hygiene instructions were given; only S5 did not provide evidence of any oral hygiene instruction. Some brushing techniques were evidenced, such as the modified-Bass technique (S4 and S8) and the modified-Stilmann technique (S7). There were also oral hygiene aids with dental floss (S8 and E9); brushes Curaprox^®^ (S7) were cited, and toothpaste was evidenced, namely, Colgate Total^®^ (S1 and S4).

### 3.2. Administration Route and Concerns

In the selection of *L. reuteri* administration, besides the strains having varied among the studies, the administration pathway varied in local (S8), topical (S4 and S9), and pills (S1, S2, S3, S5, S6, and S7). All the articles that mentioned the use of tablets, although there was a variation in the days they were taken, referred to the use of *L. reuteri* twice a day.

It should be noted that the diagnosis of periodontitis, according to the new classification of Periodontology by the AAP and EFP, encompasses various stages depending on the severity and complexity of the disease, as well as the degrees of risk progression. In the studies, there is a lack of standardization for this parameter. Individualized and selective protocols for each patient are not yet known; thus, this fact ends up being a limitation in the comparison among studies. Other factors, such as SRP sessions, also varied among the various studies; S2 and S8 showed that SRP was performed twice a week, while S3 and S6 stated that SRP was completed in 5 consultations. S1 stated that SRP was done on two consecutive days. On the other hand, S4, S5, S7, and S9 did not clarify how many times and sections the patients underwent the treatment, but only implied that SRP was performed once. All the articles considered in the present systematic review were RCTs, some were double-blinded, and only one article was a prospective split-mouth study (S9).

### 3.3. Clinical Parameters

All data are detailed in Table 3. The clinical criteria evaluated were not the same in all articles, varying between PD, BoP, IP, CAL, IG, GBI, and REC. The clinical criteria GBI (presented in S7), IG (presented in S2), and REC (presented in S1) were parameters shown only within one study, which harmed correlations. In addition, the time of “follow-up” was another characteristic that limited the correlation of the articles; they varied between 14, 40, and 90 days (1 study for each period), two after 84 days, three evaluated patients for 180 days, and one after 360 days.

After 84 days, there was a correlation only between S1 and S4. In BoP, S4 (56.58%) and S1 (55.19%) showed a reduction. Conversely, PD showed a greater decrease in S4 (41.20%), followed by a reduction of 34.22% in S1. CAL had a greater reduction in S4 (20.99%) compared to S1, with a reduction of 20.12%. Finally, the PI was not evaluated since S1 did not have PI assessed, unlike S4.

In 90 days, BoP showed the greatest reduction in S8 (100%), followed by S2 (73.25%), S9 (66.07%), S6 (32.9%), and finally S3 (22.1%). CAL in S8 had the greatest reduction and improvement (70.97%), followed by S9 (19.96%), S6 (8.02%), and finally S3 (4.96%). The PD had a greater reduction in S8 (43.14%), followed by S2 (27.34%), S6 (20.84%), S9 (15.07%), and finally S3 (12.9%). For PI, there was a greater reduction in S2 (73.8%) and S3 (19.6%). The PI showed in S8 was challenging to relate with the other articles because it followed the Greene-Vermillion debris component.

The last comparison according to the “follow-up” was conceived at 180 days. BoP had a greater reduction in S2 (76.6%), followed by S8 (60%), S6 (35.7%), and finally, S3 (29.9%). CAL showed a greater reduction in S8 (41.93%), followed by S6 (7.1%), and S3 (4.76%). PD, on the other hand, showed a greater reduction in S2 (35.37%), followed by S8 (35.42%), S6 (23.53%), and S3 (16.13%). Finally, PI showed a greater reduction in S2 (72.49%), followed by a reduction of 19.6% in S3; S8 cannot be related to S2 and S3 because it is in accordance with the Greene-Vermillion debris component, and S6 showed no results.

When analyzing the improvement (reduction) of the various clinical parameters at *t* = 84, *t* = 90, and *t* = 180 days, it was noticeable that S8 presented the greatest reductions in BoP, PD, and CAL at 90 days, and only in CAL at 180 days. It can be related to the fact this study used local application using a syringe in four moments, followed by the performance of the oral hygiene instructions (modified-Bass technique), the use of dental floss, and DSM 17938 strain only, which did not present unwanted resistance. S2 was administered in pills in contrast to S8, which used local application. It showed greater reductions in BoP and PD; therefore, the study did not show the strain used. However, oral hygiene instructions were given (no brushing technique was mentioned), as well as the use of hygiene aids. S9 presented results only for 90 days, with topical application (at 5 moments), emphasizing oral hygiene instructions and the use of dental floss to the participants, with the DSM 17938 strain; it found moderate improvement values for all clinical parameters.

### 3.4. Overall Conclusion of the Articles Included

S3 and S6 indicated oral hygiene instructions to the patients, highlighting the use of *L. reuteri* DSM 17938, ATCC PTA 5289, and in pills (twice a day), and presenting low values in reducing clinical parameters. However, S3 had the lowest values of all clinical parameters (at 90 and 180 days). Regarding S1 and S4, they were the only ones with a follow-up of 84 days, and all had clinical parameters in common. S4 showed improvements in the percentage (%) reduction compared to S1. Both had oral hygiene instructions and specified the use of Colgate Total^®^, but S4 possibly had the advantage of instructing the participants on the modified-Bass technique. Otherwise, S4 did not show the strain used, and S1 used both strains, DSM 17938 and ATCC PTA 5289.

S5 and S7 did not allow a direct comparison with other studies because the follow-up was different, even though both studies showed significant differences intergroup in all clinical parameters. S8 demonstrated that the adjuvant use of the *L. reuteri* probiotic might be a useful complement to SRP in chronic periodontitis; however, when comparing the intergroup relationship, there was no significant difference between the test and control groups. Conversely, S2 concluded that the adjuvant use of *L. reuteri* (pill) improved clinical and microbiological outcomes in chronic periodontitis, with significant intergroup improvements in all parameters and at all assessment time points.

The adjuvant use of probiotics associated with SRP had no additional clinical efficacy when compared to SRP alone (S3), whereas S4 obtained significant reductions in both groups, with greater improvement in the test group; only PI had no significant result. This study states that probiotics can be used as an adjuvant to the SPR with superior results compared to SRP alone. In the CAL parameter, S5 showed there was a decrease in the values for both groups, but the intergroup relationship was statistically significant in favor of the test group; thus, S5 revealed that the use of *L. reuteri* in pills, along with SRP, was effective.

S6 evaluated BoP, PI, CAL, and PD. Both CAL and PD showed a higher level of significance between groups at 90 days. Similarly, S7 had a statistically significant level in both groups for the clinical parameters (BoP, PI, CAL, and GBI) with a higher intergroup significance in the test group; the authors demonstrated that probiotics are optimal adjuvants to SRP in stage IV periodontitis. In a longer-period evaluation, 180 days, S8 presented that *L. reuteri* (local administration) showed significant results, with intergroup improvements evident in all clinical parameters (except for PI, where there was no significant difference at 180 days). The last study, S9, obtained significant reductions in both groups, with a higher significance level for the test group for all clinical parameters evaluated, particularly in CAL and BoP, for stage III and IV periodontitis patients.

### 3.5. Statistical Significance Found

Six out of 9 studies found statistically significant results for the use of the probiotic *L. reuteri*. Tekce et al. (2015) and Sufaru et al. (2022) found significant intragroup improvement in both groups for the clinical parameters PI, GI, BOP, and PD (*p* = 0.001). In addition, Tekce et al. (2015) found significant improvement for those same parameters and at all intergroup assessment periods, favoring the test group (PD [*p* = 0.001] and PI, GI, and BOP [*p* < 0.05]). Likewise, Sufaru et al. (2022) presented significant intergroup reductions at 90 and 180 days for all clinical parameters (*p* < 0.001). Similar results were reported by Hadzic et al., who showed significant intergroup reductions for GBI, CAL, and BOP in favor of the test group (respectively, *p* < 0.0001; *p* = 0.0169; *p* < 0.0001); otherwise, only PI did not have significant intergroup reductions (*p* = 0.054). Even though Sinulingga et al. (2020) evaluated only CAL, there was a significant intragroup reduction (*p* < 0.004, in both groups) and intergroup reduction (*p* < 0.05, favoring the test group). Ikram et al. (2019) found statistically significant reductions in both groups (*p* = 0.001) for the clinical parameters PD, CAL, BoP, and IP; whereas, for the intergroup comparison, PD had intergroup reductions at 42 days (*p* = 0.01) and CAL, IP, and BOP (*p* = 0.001); at 84 days, PD, CAL, and BOP showed statistically significant intergroup reductions (*p* = 0.001), whereas IP did not show intergroup improvement (*p* = 0.18). In El Bagoory et al.’s (2021) study, PI at 180 days had no statistically significant intergroup difference; otherwise, regarding BoP, there were intergroup improvements in favor of the test group at 90 days (*p* = 0.003) and 180 days (*p* = 0.011); PD showed an intergroup improvement in favor of the test group at 90 days (*p* = 0.022) and 180 days (*p* = 0.001); and CAL had intergroup improvement for the test group in both periods (90 days, *p* = 0.022; and 180 days, *p* = 0.001).

On the other hand, three studies did not find intergroup differences in the clinical findings. Pelekos et al. (2019) showed, for all clinical parameters evaluated (CAL, PD, BOP, and PI), in both groups, no significant intergroup reduction (*p* > 0.05). Similarly, Teughels et al. (2013) had no significant reductions (*p* > 0.05) for PD, CAL, REC, and BOP. Pelekos et al. (2020) reported significant intergroup reductions at 90 days favoring the test group for PD (*p* = 0.002) and CAL (*p* = 0.02), whereas BOP did not show significant reductions (*p* = 0.12); otherwise, after 180 days, no significant differences were observed for PD (*p* = 0.07), CAL (*p* = 0.09), and BOP (*p* = 0.07), showing that in a greater period, the product was not favorable.

### 3.6. Quality Assessment

**Figure 2:**
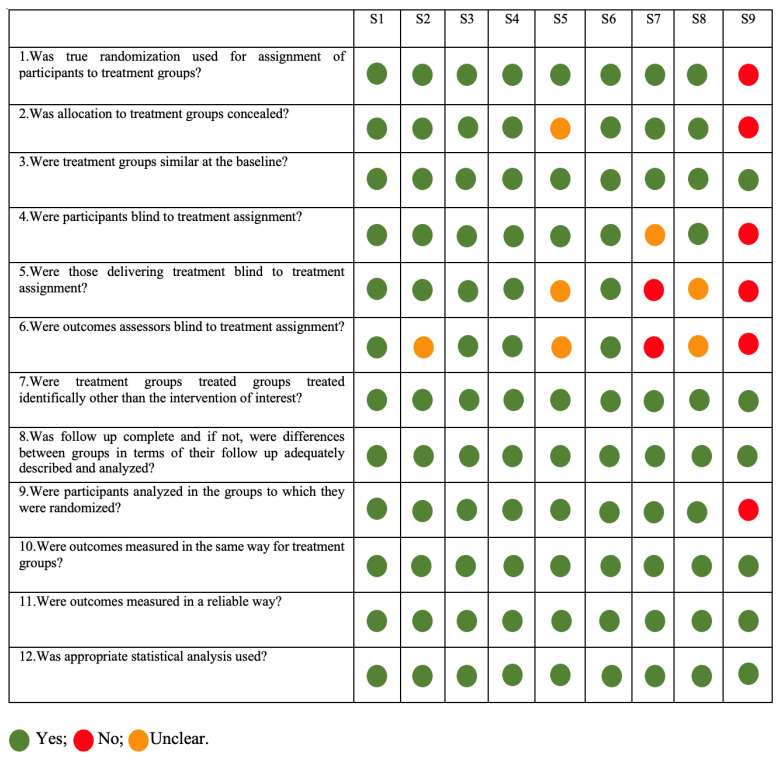
Risk of Bias analysis according to Joanna Briggs Institute critical appraisal. All green (yes) or at maximum 2 unclear—Low risk; Moderate risk of bias if one “no” (red) was found or up to 4 “unclear” criteria were met; and High risk of bias if at least 2 “no” (red) or ≥5 “unclear” were found.

The studies were evaluated, and the characteristics of the articles are presented in Figure 2. Only 5 studies had a low risk of bias (S1, S2, S3, S4, S6, and S8); S5 was the only one with moderate risk; and S7 and S9 had a high risk of bias.

## 4. Discussion

This systematic review aimed to understand the efficacy of the *Lactobacillus reuteri* (*L. reuteri*) as an adjuvant in nonsurgical periodontal treatment. It included 9 articles that demonstrated the influence of that specific probiotic in patients who underwent nonsurgical treatment of periodontitis. Probiotics were put forward in 1965 [28] and are currently considered by WHO and the Food and Agriculture Organization of the United States (FAO) as a beneficial product that affects all body systems. They have been demonstrated to play a role in maintaining the health of the urogenital system, and fighting against cancers, diabetes, obesity, and allergies [29,30,31,32,33]. Recently, studies have explored using probiotics as adjuncts in oral disease treatments. The results showed improved oral health in diseases such as caries, periodontal diseases, candida infection, and halitosis [34,35,36,37]. This fact can be observed only in 6 (66.7%) of the articles included in this study; on the other hand, 3 out of the 9 (33.3%) articles included in this systematic review did not consider improvements comparing the use of the probiotic or the nonsurgical treatment only.

Probiotics are concentrated in Lactobacillus, Streptococcus, Bifidobacterium, Weissella, and scattered species such as *Bacillus subtilis* and *Saccharomyces cerevisiae*. Thereby, strains of *Lactobacillus reuteri*, *Lactobacillus brevis*, and *Streptococcus salivarius* isolated from the oral cavity have been commercially produced and used [38,39]. It is known that more than 700 bacterial species are colonizing patient’s mouths [40], and only a few initiate and advance periodontal diseases, such as *P. gingivalis*, *Aggregatibacter actinomycetemcomitans*, *Tannerella forsythia*, *Prevotella intermedia*, and *Fusobacterium nucleatum* [41]. Articles revealed that probiotics could effectively inhibit periodontopathogens and improve clinical parameters related to periodontal health and inflammation biomarkers. The short- and long-term probiotic effects were assessed when they were used as an adjunctive for nonsurgical periodontal treatment. Kuru et al. (2017) [42] evaluated 51 healthy periodontal volunteers who were randomized into two groups which received either yogurt containing placebo or *Bifidobacterium animalis* subsp. lactis DN-173010 for 28 days, followed by 5 days of non-brushing. In the group of probiotic use, all parameters had significant improvement after 33 days, leading to the knowledge that short-term use positively affects plaque accumulation and gingival inflammatory parameters. This fact was observed in many of the articles included in this review, agreeing with positive short-term results. When evaluated in the long-term [34], 30 patients with chronic periodontitis who received *L. reuteri*-containing lozenge or placebo (twice a day) after SRP, the results showed that the probiotic group’s clinical parameters were better than the placebo, at all time points, mainly from day 90, presenting significant higher CAL gain. This fact was not a common result within all studies included here, some of which had no efficacy in the long term.

In another meta-analysis study [43], the authors assessed the effects of probiotics after nonsurgical periodontal treatment for 42–360 days. They concluded probiotics could help to significantly reduce CAL in moderately deep pockets. Negatively, 3 out of 4 of the included studies showed significant heterogeneity. Therefore, studies suggested that short- and long-term applications of probiotics improved clinical parameters. Our results in the present systematic review partially agree with the studies mentioned above because 3 out of 9 articles did not improve by using the probiotic; therefore, the other 6 studies showed a significant and better clinical outcome for the test group (use of probiotic *L. reuteri*).

The choice to study *Lactobacillus reuteri* came from many other studies which proved its inhibitory effects on periodontopathogens, including *P. gingivalis* [44]. It may be attributed to its specific by-product, a non-protein broad-spectrum antibiotic named reuterin, which suppresses the growth of many gram-positive/negative bacteria, yeast, and fungi [45]. Both live *L. reuteri* ATCC PTA 5289 and DSM 17938—observed in three studies included in this systematic review (S7, S8, and S9)—showed inhibition of *P. gingivalis*. In contrast, only the live form of the two *L. reuteri* attenuated the *A. actinomycetemcomitans* growth in vitro [46,47]. Another subspecies, *L. reuteri* ATCC 55730, also inhibited the growth of *F. nucleatum* ATCC 10953, *P. gingivalis* ATCC 33277, and *A. actinomycetemcomitans* ATCC 33384. They also protected cells infected by periodontal pathogens from death [48]. Moreover, *L. reuteri* DSM 17938 produces exopolysaccharide, which benefits its adhesion to epithelial cells, competing with pathogenic bacteria for adhesion sites [49]. Kang et al. (2011) [50] demonstrated that *L. reuteri* KCTC 3594 inhibited the secretion of IL-6 induced by *F. nucleatum*, causing a modulation in the inflammatory response.

Pre-clinical experiments showed the live *L. reuteri* DSM 17938 and PTA 5289 could upregulate immune responses [46,47]. In clinical studies, similarly to the RCTs in our systematic review, the use of *L. reuteri* showed the inhibition of *P. gingivalis* and *P. intermedia* in saliva, and *P. gingivalis* in the supra and subgingival plaque [51]. However, for peri-implant mucositis, a disease not included in this review, *L. reuteri* DSM 17938 and PTA 5289 reduced the load of *P. gingivalis* [52]. *L. reuteri* also caused immunomodulatory effects such as regulating the imbalance between MMP and TIMP [34] and reducing the production of TNF-a, IL-1b, and IL-17 (pro-inflammatory cytokines) [18], contributing to relieving the inflammatory response and reducing periodontal tissues destruction.

Despite the strict inclusion and exclusion criteria, this systematic review presented some limitations in the correlation of the articles. Although it focused only on the probiotic *L. reuteri*, there was a difference in the strains of *L. reuteri* throughout the analysis of all studies. We know the DSM 17938 strain did not show unwanted resistance; on the other hand, the ATCCPTA 5289 strain exhibited resistance [27], which leads us to understand the results may be altered depending on the strain applied. S5, S8, and S9 used the DSM 17938 strain only in pills, local, and topical forms. S1, S3, S6, and S7 used *L. reuteri* DSM 17938 and ATCC PTA 5289 strains. Otherwise, S2 and S4 did not show which strain(s) were used.

Moreover, the administration pathway varied in local (S8), topical (S4 and S9), and pills (S1, S2, S3, S5, S6, and S7), which can interfere with the results obtained, harming comparisons. The fact that the dosage of *L. reuteri* was not stated can be another limitation since the dose administered for different diagnoses (gingivitis or periodontitis) should be adjusted for each patient. The articles included different ages, ranging from 18 to 56 years, which can be another fundamental factor of comparison causing bias in the interpretation of the results; moreover, the number of included patients varied between 12 and 40. Furthermore, the clinical criteria GBI, IG, and REC were parameters shown only within one study, and the time of “follow-up” was different among the studies, which impaired correlations.

## 5. Conclusions

Within the limitations of this study and considering the heterogeneity among the studies, the use of *L. reuteri* as an adjunct to nonsurgical periodontal treatment may result in significantly better clinical outcomes than nonsurgical periodontal treatment alone, confirming the positive hypothesis raised. It is also important to emphasize oral hygiene procedures, respecting the appropriate brushing techniques and the interdental aids used for plaque removal.

More RCTs with larger sample sizes, longer follow-ups, and a standardized, individualized, and selective protocol for *L. reuteri* administration are needed to determine the optimal dose, frequency, and duration of the probiotic use. Furthermore, studies need to evaluate better microbiological parameters to perceive “microbiological change” and *L. reuteri* colonization. In addition, it is essential to highlight that narrowing down the analysis on a specific probiotic (*L. reuteri*) brings little advancement to the microbial complex, salivary, and serum metabolomics discussion, even though it presented interesting results for periodontal parameters. Thus, we suggest new studies considering the analysis of all above-mentioned parameters, including more probiotics, to compare results, efficacy, and metabolomics status.

## Figures and Tables

**Table 1:** PICO strategy developed.

P (Population)	Patients with periodontal disease
I (Intervention)	Administration of the probiotic *L. reuteri* as an adjunct to the nonsurgical periodontal treatment
C (Comparison)	Compare results of administering probiotic *L. reuteri* adjuvant to nonsurgical periodontal treatment compared to nonsurgical treatment alone.
O (Results)	Possible reduction of clinical parameters (PD, BOP, PI, CAL, GI, GBI, and REC) when associated with the probiotic *L. reuteri* to nonsurgical periodontal treatment.

**Table 2:** Eligibility criteria.

Inclusion Criteria	Exclusion Criteria
– Published in the last 10 years	– Species other than humans
– English language	– in vitro studies
– Full text available	– Reviews and meta-analyses
– Clinical Trials, Randomized Controlled Clinical Trials, Controlled Clinical Trials, Double-blind clinical trials, Human Studies	– Studies including smokers or patients with systemic disease

**Table 3:** Characteristics of the studies included in the systematic review.

Author/Year	Study Design	Objective	Patients (*n*)	Age/Gender	Periods of Evaluation	Administration of *L. reuteri*, CFU, and Strain	Clinical Parameters Evaluated	Initial Evaluation (Baseline)	Final Evaluation	Other Evaluations	*p* (Intergroup Significance Value)
**S1** (Teughels et al., 2013)	Double-blind, “parallel-arm” RCT	Evaluate the clinical and microbiological effects of l reuteri (lozenge) as an adjuvant to SRP compared to SRP plus placebo	30	>18 years 15 F/15 M	*t* = 0 days *t* = 21 days *t* = 42 days *t* = 63 days *t* = 84 days	Pill (2× a day for 84 days); (1 × 10^8^ CFU); DSM 17938 and ATCC PTA5289 (Prodentis; BioGaia, Lund, Sweden)	PD, CAL, REC, BoP	**CG** CAL: 4.97 ± 0.61 mm; PD: 4.32 ± 0.50 mm; REC: 0.66 ± 0.73 mm; BoP: 67.53 ± 11.37% **TG** CAL: 4.97 ± 1.01 mm; PD: 4.15 ± 0.71 mm; REC: 0.82 ± 0.71 mm; BoP: 70.70 ± 14.53%;	**CG** CAL: 4.21 ± 0.67 mm; PD: 2.93 ± 0.40 mm; REC: 1.28 ± 0.42 mm; BoP: 16.58 ± 10.54% **TG** CAL: 3.97 ± 0.97 mm; PD: 2.73 ± 0.57 mm; REC: 1.24 ± 0.75 mm; BoP: 15.51 ± 11.92%;	N/R	**PD** t = 0 days (*p* > 0.05); t = 21 days, t = 42 days, t = 63 days (*p* N/R); t = 84 days (*p* = 0.097); **CAL** t = 0 days (*p* > 0.05); t = 21 days, t = 42 days, t = 63 days (*p* N/R); t = 84 days (*p* > 0.05); **REC** t = 0 days (*p* > 0.05); =21 days, t = 42 days, t = 63 days (*p* N/R); t = 84 days (*p* > 0.05); **BoP** t = 0 days (*p* > 0.05); =21 days, t = 42 days, t = 63 days (*p* N/R); t = 84 days (*p* > 0.05)
**S2** (Tekce et al., 2015)	Parallel and double-blind RCT	To evaluate the effect of *L. reuteri* (clinical and microbiological) as an adjunctive treatment to SRP for patients with chronic periodontitis.	40	18–22 years 22 F/18 M	*t* = 0 days *t* = 21 days *t* = 90 days *t* = 180 days *t* = 360 days	Pill (2× a day for 28 days); (1 × 10^5^ CFU); *L. reuteri*- containing lozenges (Prodentis, BioGaia, Lund, Sweden)	BoP, PI, GI, PD	**CG** GI: 2.12 ± 0.21%; PD: 5.36 ± 0.72 mm; PI: 2.30 ± 0.41%; BoP: 88.65 ± 4.11%; **TG** GI: 2.12 ± 0.15%; PD: 5.23 ± 0.68 mm; PI: 2.29 ± 0.28%; BoP: 88.90 ± 7.66%	**CG** GI: 1.66 ± 0.36%; PD: 4.80 ± 0.70 mm; IP: 1.39 ± 0.28%; BoP: 19.05 ± 4.84%; **TG** GI: 0.80 ± 0.38%; PD: 3.49 ± 0.87 mm; PI: 0.73 ± 0.24%; BoP: 11.05 ± 3.99%	**CG** GI: t = 21 days 1.34 ± 0.48%, t = 90 days 1.53 ± 0.48%, t = 180 days 1.54 ± 0.35%; PD: t = 21 days 4.60 ± 0.71 mm, t = 90 days 4.51 ± 0.71 mm, t = 180 days 4.66 ± 0.69 mm; PI: t = 21 days 0.93 ± 0.41%, t = 90 days 1.14 ± 0.29%, t = 180 days 1.23 ± 0.35%; BoP: t = 21 days 25.65 ± 4.75%, t = 90 days 21.85 ± 3.98%, t = 180 days 19.95 ± 4.88%; **TG** GI: t = 21 days 0.61 ± 0.28%, t = 90 days 0.76 ± 0.35%, t = 180 days 0.69 ± 0.37%; PD: t = 21 days 4.03 ± 0.74 mm, t = 90 days 3.80 ± 0.75 mm, t = 180 days 3.38 ± 0.86 mm; PI: t = 21 days 0.48 ± 0.17%, t = 90 days 0.60 ± 0.21%, t = 180 days 0.63 ± 0.24%; BoP: t = 21 days 21.50 ± 5.88%, t = 90 days 16.65 ± 4.21%, t = 180 days 12.30 ± 4.82%	**GI** t = 0 days (*p* > 0.05); t = 21 days (*p* **< 0.005**); t = 90 days (*p* **< 0.005**); t = 180 days (*p* < 0.005); t = 360 days (*p* **< 0.005**). **PD** t = 0 days (*p* > 0.05); t = 21 days (*p* **= 0.001**); t = 90 days (*p* **= 0.001**); t = 180 days (*p* **= 0.001**); t = 360 days (*p* **= 0.001**). **PI** t = 0 days (*p* > 0.05); t = 21 days (*p* **< 0.005**); t = 90 days (*p* **< 0.005**); t = 180 days (*p* **< 0.005**); t = 360 days (*p* **< 0.005**). **BoP** t = 0 days (*p* **> 0.05**); t = 21 days (*p* **< 0.005**); t = 90 days (*p* **< 0.005**); t = 180 days (*p* **< 0.005**); t = 360 days (*p* **< 0.005**).
**S3** (Pelekos et al., 2019)	Double-blind parallel RCT	To evaluate the efficacy of the probiotic *L. reuteri* as an adjuvant to SRP in treating periodontal disease.	41	52.3 average age, >35 years 26 F/15 M	*t* = 0 days *t* = 90 days *t* = 180 days	Pill (2× a day for 28 days) (1 × 10^8^ CFU); ATCC PTA5289 (Prodentis, Biogaia, Sweden)	BoP, PI, CAL, PD	**CG** CAL: 4.9 ± 1.7 mm; PD: 3.5 ± 1.0 mm; PI: 52.8 ± 24.8%; BoP: 69.1 ± 27.8%; **TG** CAL: 4.2 ± 1.3 mm; PD: 3.1 ± 0.6 mm; PI: 41.9 ± 23.7%; BoP: 59.5 ± 21.3%	**CG** CAL: 4.6 ± 1.6 mm; PD: 2.9 ± 0.6 mm; PI: 23.7 ± 16.7%; BoP: 36.7 ± 17.1%; **TG** CAL: 4.0 ± 1.3 mm; PD: 2.6 ± 0.4 mm; PI: 22.3 ± 13,7%; BoP: 29.6 ± 12.1%	**(90 days)** **CG** CAL: 4.6 ± 1.6 mm; PD: 3.0 ± 0.6 mm; PI: 29.7 ± 21.2%; BoP: 42.2 ± 17.6%; **TG** CAL: 4.0 ± 1.3 mm; PD: 2.7 ± 0.5 mm; PI: 26.5 ± 15.1%; BoP: 37.4 ± 20.1%	**BoP** t = 0 days (*p* = 0.215); t = 90 days (*p* = 0.434); t = 180 days (*p* = 0.180); **PI** t = 0 days (*p* = 0.163); t = 90 days (*p* = 735); t = 180 days (*p* = 0.99); **CAL** t = 0 days (*p* = 0.241); t = 90 days (*p* = 0.175); t = 180 days (*p* = 0.167); **PD** t = 0 days (*p* = 0.230); t = 90 days (*p* = 0.141); t = 180 days (*p* = 0.246)
**S4** (Ikram et al., 2019)	Double-blind RCT	To evaluate the clinical efficacy of SRP alone and SRP together with an adjuvant probiotic containing *L. reuteri* in the treatment of chronic periodontitis and to compare the efficacy of the two treatments	28	N/I age 11 F/17 M	*t* = 0 days *t* = 42 days *t* = 84 days	Topical application, in paste (mixture of water and powder) (2× a day for 84 days); (CFU = not reported); Not reported	BoP, PI, CAL, PD	**CG** CAL: 4.12 ± 0.74 mm; PPD: 4.25 ± 1.12 mm; IP: 84.58 ± 8.06%; BoP: 71.94 ± 23.13%; **TG** CAL: 4.08 ± 0.66 mm; PPD: 4.32 ± 0.91 mm; IP: 85.23 ± 8.23%; BoP: 70.47± 11.8%	**CG** CAL: 3.86 ± 0.59 mm; PPD: 3.95 ± 0.78 mm; IP: 33.67 ± 9.47%; BoP: 46.24 ± 11.40%; **TG** CAL: 3.24 ± 0.47 mm; PPD: 2.54 ± 0.52 mm; IP: 26.28 ± 4.12%; BoP: 13.89 ± 3.25%	**(42 days)** **CG** CAL: 3.99 ± 0.89 mm; PPD: 4.08 ± 0.76 mm; IP: 54.38 ± 8.13%; BoP: 58.23± 12.77%; **TG** CAL: 3.69 ± 0.67 mm; PPD: 3.44 ± 0.64 mm; IP: 43.46 ± 9.17%; BoP: 34.25± 6.32%	**BoP** t = 0 days (*p* > 0.05); t = 42 days (*p* **= 0.001**); t = 84 days (*p* **= 0.001**); **PI** t = 0 days (*p* > 0.05); t = 42 days (*p* **= 0.001**); t = 84 days (*p* = 0.18); **CAL** t = 0 days (*p* > 0.05); t = 42 days (*p* **= 0.001**); t = 84 days (*p* **= 0.001**); **PD** t = 0 days (*p* > 0.05); t = 42 days (*p* **= 0.01**); t = 84 days (*p* **= 0.001**)
**S5** (Sinulingga et al., 2020)	RCT	To evaluate the effect of *L. reuteri* on CAL and IL-4 levels in patients with periodontitis after SRP	16	20–56 years N/I for gender	*t* = 0 days *t* = 14 days	Pill for 14 days. N/I for the number of times used; (CFU = not reported); DSM 17938	CAL	**CG** CAL: 6.70 ± 0.82 mm; **TG** CAL: 6.70 ± 0.82 mm	**CG** CAL: 4.70 ± 0.67 mm; **TG** CAL: 3.90 ± 1.37 mm	N/A	**CAL** t = 0 days (*p* > 0.05); t = 14 days (*p* **< 0.05**)
**S6** (Pelekos et al., 2020)	Double-blind “parallel arms” RCT	To evaluate the effects of the probiotic *L. reuteri* as an adjuvant with placebo on molars with deep pockets	40	Average of 52 years26 F/14 M	*t* = 0 days *t* = 90 days *t* = 180 days	Pill (2× a day for 28 days); (1 × 10^8^ CFU); DSM 17938 and ATCC PTA5289 (Prodentis, Biogaia, Sweden)	BoP, PI, CAL, PD	**CG** CAL: 8.02 ± 2.32 mm; PPD: 6.38 ± 1.68 mm; IP: S/R BoP: 221(93.2%) **TG** CAL: 7.61 ± 1.99 mm; PD: 5.95 ± 1.19 mm	**CG** CAL: 7.50 ± 2.58 mm; PPD: 4.97 ± 1.91 mm; IP: S/R BoP: 145(61.2%); **TG** CAL: 7.07 ± 2.20 mm; PD: 4.55 ± 1.37 mm; IP: S/R BoP: 110(52.4%)	**(90 days)** **CG** CAL: 7.59 ± 2.53 mm; PPD: 5.30 ± 1.92 mm; IP: S/R BoP: 149(62.9%); **TG** CAL: 7.00 ± 2.20 mm; PPD: 4.71 ± 1.41 mm; IP: S/R BoP: 116 (55.2%)	**BoP** t = 0 days (*p* = 0.09); t = 90 days (*p* = 0.12); t = 180 days (*p* = 0.07) **PI** N/R **CAL** t = 0 days (*p* = 0.12); t = 90 days (*p* **= 0.02**); t = 180 days (*p* = 0.09); **PD** t = 0 days (*p* = 0.07); t = 90 days (*p* **= 0.002**); t = 180 days (*p* = 0.07)
**S7** (Hadžić et al., 2021)	RCT	To evaluate the effects of *L. reuteri* pills (DMS 17938 and ATCC PTA 5289) as an adjuvant therapeutic agent in combination with root scraping and smoothing	40	35–50 years 21 F/19 M	*t* = 0 days *t* = 40 days	Pill (2× a day for 40 days); (1 × 10^4^ CFU); DMS 17938 and ATCC PTA 5289	BoP, PI, CAL, GBI	**CG** CAL: 6.1 ± 1.3 mm; BoP: 73.2 ± 25.5%; IP: 2.3 ± 0.71%; GBI: 85.1 ± 13.8% **TG** CAL: 5.8 ± 1.9 mm; BoP: 66.7 ± 23.1%; PI: 2.1 ± 0.63%; GBI: 83.3 ± 16.2%	**CG** CAL: 5.6 ± 0.8 mm; BoP: 31.9 ± 9.8%; IP: 0.34 ± 0.22%; GBI: 35.1 ± 13.5% **TG** CAL: 4.1 ± 0.4 mm; BoP: 49.2 ± 10.1%; PI: 0.55 ± 0.23%; GBI: 67.5 ± 12.3%	N/A	**BoP** t = 0 days (*p* = 0.4035); t = 40 days (*p* **< 0.001**); **PI** t = 0 days (*p* = 0.3520); t = 40 days (*p* **= 0.0054**); **CAL** t = 0 days (*p* = 0.5635); t = 40 days (*p* **= 0.0169**); **GBI** t = 0 days (*p* = 0.7073); t = 40 days (*p* **< 0.001**)
**S8** (El-Bagoory et al., 2021)	RCT	To determine the additional benefit of *L. reuteri* (DSM 17938) to SRP in the treatment of periodontal disease concerning clinical and microbiological parameters	12	35–55 years 3 F/9 M	*t* = 0 days *t* = 90 days *t* = 180 days	Local, in a syringe (0, 7, 14, and 28 days); (1 × 10^8^ CFU); DSM 17938 (silicon dioxide) (BioGaia, Lund, Sweden)	BoP, PI, CAL, PD	**CG** CAL: 3.30 ± 0.48 mm; PPD: 5.30 ± 0.48 mm; IP: 100% (>2/3 of exposed tooth surface). BoP: 100% **TG** CAL: 3.10 ± 0.32 mm; PPD: 5.10 ± 0.32 mm; IP: 100% (>2/3 of exposed tooth surface); BoP: 100%	**CG** CAL: 2.30 ± 0.67 mm; PPD: 4.30 ± 0.67 mm; IP: 50% (>1/3 of exposed tooth surface) e 50% (>2/3 of exposed tooth surface); BoP: 100% **TG** CAL: 1.30 ± 0.48 mm; PPD: 3.30 ± 0.48 mm; IP: 70% (>1/3 of exposed tooth surface) and 30% (>2/3 of exposed tooth surface);BoP: 40%	**(90 days)** **CG** CAL: 1.50 ± 0.71 mm;PPD: 3.50 ± 0.71 mm; PI: 50% (<1/3 of exposed tooth surface) e 50% (>1/3 of exposed tooth surface); BoP: 70% **TG** CAL: 0.90 ± 0.32 mm; PPD: 2.90 ± 0.32 mm; PI: 100% (<1/3 of exposed tooth surface); BoP: 0%	**CAL** t = 0 days (*p* = 0.276); t = 90 days (*p* **= 0.022**); t = 180 days (*p* **= 0.001**); **PD** t = 0 days (*p* = 0.276); t = 90 days (*p* **= 0.022**); t = 180 days (*p* **= 0.001**); **PI** t = 0 days (*p* = 1.000); t = 90 days (*p* **= 0.033**); t = 180 days (*p* = 0.650); BoP t = 0 days (*p* = 1.000); t = 90 days (*p* = 0.003); t = 180 days (*p* **= 0.011**)
**S9** (Sufaru et al., 2022)	Prospective split-mouth study	Evaluate the clinical effects with local application of *L. reuteri* (DSM 17938) in periodontal pockets in severe periodontitis	40	48.65 average age 21 F/19 M	*t* = 0 days *t* = 90 days	Topical (2 quadrants per patient), at 5 times (0, 7, 14, 21, and 28 days); (1 × 10^8^ CFU); DSM 17938 (Protectis^®^, BioGaia, Stockholm, Sweden)	BoP, CAL, PD	**CG** CAL: 5.02 ± 0.65 mm; PPD: 6.09 ± 0.51 mm; BoP: 81.67 ± 6.5%; **TG** CAL: 4.96 ± 0.63 mm PPD: 6.04 ± 0.42 mm BoP: 80.90 ± 6.35%	**CG** CAL: 4.65 ± 0.62 mm; PPD: 5.58 ± 0.49 mm; BoP: 26.40 ± 9.54; **TG** CAL: 3.97 ± 0.65 mm; PPD: 5.13 ± 0.54 mm; BoP: 14.92 ± 6.17%	N/A	**BoP** t = 0 days (*p* = 0.595); t = 90 days (*p* **< 0.001**); **CAL** t = 0 days (*p* = 0.650); t = 90 days (*p* **< 0.001**); **PD** t = 0 days (*p* = 0.650); t = 90 days (*p* **< 0.001**)

RCT: randomized controlled trial; F: female; M: male; N/A: Not applicable; N/R: No results; N/I: Not identified; Statistically significant results marked in bold; CG: control group; TG: test group; PD: pocket depth; GI: gingival index; CAL: clinical attachment level; BoP: bleeding on probing, PI: Plaque index; CFU: Colony forming unit.
